# Genomic copy number and expression patterns in testicular germ cell tumours

**DOI:** 10.1038/sj.bjc.6604079

**Published:** 2007-12-04

**Authors:** A McIntyre, B Summersgill, Y J Lu, E Missiaglia, S Kitazawa, J W Oosterhuis, L H Looijenga, J Shipley

**Affiliations:** 1 Molecular Cytogenetics, Section of Molecular Carcinogenesis, The Institute of Cancer Research, Sutton, Surrey, UK; 2 Department of Molecular Pathology, Kobe University School of Medicine, Chuo-ku, Kobe, Japan; 3 Department of Pathology, Erasmus MC-University Medical Center Rotterdam, Daniel den Hoed Cancer Center, Josephine Nefkens Institute, Rotterdam, The Netherlands

**Keywords:** testicular germ cell tumour, spermatocytic seminoma, chromosome, comparative genomic hybridisation, CESH

## Abstract

Testicular germ cell tumours of adults and adolescents (TGCT) include seminomas (SE) and nonseminomas (NS), with spermatocytic seminomas (SSE) representing a distinct entity in older men. SE and NS have gain of 12p material in all cases, whereas SSE are associated with overrepresentation of chromosome 9. Here, we compare at the chromosomal level, copy number imbalances with global expression changes, identified by comparative expressed sequence hybridisation analyses, in seven SE, one combined tumour, seven NS and seven cell lines. Positive correlations were found consistent with copy number as a main driver of expression change, despite reported differences in methylation status in SE and NS. Analysis of chromosomal copy number and expression data could not distinguish between SE and NS, in-keeping with a similar genetic pathogenesis. However, increased expression from 4q22, 5q23.2 and 9p21 distinguished SSE from SE and NS and decreased copy number and expression from 2q36–q37 and 6q24 was a specific feature of NS-derived cell lines. Our analysis also highlights 19 regions with both copy number and expression imbalances in greater than 40% of cases. Mining available expression array data identified genes from these regions as candidates for involvement in TGCT development. Supplementary data is available at http://www.crukdmf.icr.ac.uk/array/array.html.

Adult germ cell tumours of the testis can be grouped by age of incidence and differentiation status into two main entities (Oosterhuis and Looijenga, 2005). Firstly, testicular germ cell tumours (TGCT) of adults and adolescents. These are the most common malignancy in males aged 20–40 years (Horwich *et al*, 1991) accounting for 60% of all male malignancies in this age group (Ulbright, 1993). TGCT can be classified into two main subtypes, seminoma (SE) and nonseminoma (NS). SE have a median age at diagnosis of 35 and resemble primordial germ cells (PGC)/gonocytes, the cells from which both these subtypes are generally thought to be derived. NS, which have a median age at diagnosis of 25, exhibit various stages of differentiation with cells from embryonic, extra-embryonic and somatic tissue types (Mostofi, 1973). Material from the short arm of chromosome 12 is invariably gained in these tumours and in roughly 10% of cases a smaller region of gain at 12p11.2–p12.1 has been defined (Rodriguez *et al*, 2003; Zafarana *et al*, 2003).

Secondly, spermatocytic seminomas (SSE) are also testicular tumours of germ cell origin, however, they are generally restricted to men aged over 50 years (Oosterhuis and Looijenga, 2005). The genomic imprinting in SSE has a paternal pattern that is different to that of SE and NS, which generally show an erased pattern of imprinting. This is consistent with SSE being derived from a more differentiated germ cell in the spermatogenic lineage than SE or NS (Verkerk *et al*, 1997). Unlike the TGCT of adults and adolescents, gain of 12p material is not a feature of these tumours. However, gain of chromosome 9 is associated with SSE and array comparative genomic hybridisation analysis identified amplification of 9p21.3-ter and the likely involvement of the *DMRT1* gene (Looijenga *et al*, 2006).

Cytogenetic, comparative genomic hybridisation (CGH) and allelic imbalance studies of SE and NS have shown that a number of regions in addition to 12p are imbalanced. Reported frequent aberrations common to both SE and NS include gain of material from chromosomes 1, 7, 8, 17, 21 and X and loss of material from chromosomes 4, 5, 11, 13, 18 and Y (van Echten *et al*, 1995; Korn *et al*, 1996; Mostert *et al*, 1996; Sandberg *et al*, 1996; Summersgill *et al*, 1998a, 1998b; Kraggerud *et al*, 2002; von Eyben *et al*, 2004). In recent years, a number of expression profiling studies have been reported and these have identified overexpressed genes and expression signatures, which distinguish different subtypes of NS (Skotheim *et al*, 2005). Although we have previously examined gene copy number and expression changes associated with 12p (Rodriguez *et al*, 2003), few studies have integrated genomic and expression array data for the entire genome of TGCT (Skotheim *et al*, 2006). Factors predicted to influence gene expression include genomic copy number and epigenetic factors such as methylation. The level of methylation in TGCT varies between the subtypes and previous studies have found that SE have a lower level of CpG island methylation than NS (Smiraglia *et al*, 2002; Zhang *et al*, 2005).

Here, we investigate a series of SSE, SE, NS and cell lines using the approach of comparative expressed sequence hybridisation (CESH) (Lu *et al*, 2001) to determine a global chromosomal level view of the expression patterns in these tumours. This and CGH data are analysed to determine the relationship between expression patterns and copy number imbalances. In conjunction with published data for SE and NS, regions of imbalance associated with differentially expressed genes in greater than 40% of cases were identified. In addition, changes in expression are identified which differentiate between TGCT, NS-derived cell lines and SSE.

## MATERIALS AND METHODS

Snap-frozen primary tumour samples were collected from histologically verified SE (26), NS (24), combined tumours (CT) (6) and SSE (5) obtained from patients attending the Royal Marsden Hospital NHS Trust and Erasmus MC University Medical Center, Rotterdam (Supplementary Table 1). Informed patient consent and ethical approval were obtained for the use of the material in this study in accordance with the Helsinki Declaration. In addition, nine cell lines derived from NS tumours and one derived from a SE were also included in the study. These were GCT27, GCT44, Tera1, Tera2, H12.2, 2102Ep, 577MF, NTERA2, SUSA and TCam-2. The cell lines were cultured as described previously (Pera *et al*, 1987; Kelland *et al*, 1992; Mizuno *et al*, 1993; Summersgill *et al*, 2001; Goddard *et al*, 2007). A pool of 45 individual samples of normal testis RNA was available from Clontech (Mountain View, CA, USA) and normal DNA from Sigma (St Louis, MO, USA). Information as to which samples were studied by CGH and CESH can be found in Supplementary Table 1 at http://www.crukdmf.icr.ac.uk/array/array.html.

### Comparative genomic hybridisation

CGH was performed as described previously (Summersgill *et al*, 1998b) using genomic DNA prepared by standard procedures from patient tissue, cell lines, together with normal reference DNA. Images were captured using a cooled CCD Photometrics camera with SmartCapture software (Digital Scientific, Cambridge, UK). Image analysis was carried out using Quips-XL software (Vysis Inc., Des Plaines, IL, USA). At least five representative images were fully analysed and the results from these were studied separately and also combined to produce an average fluorescence ratio for each chromosome. A copy number change was indicated when the average fluorescence ratio lay outside the normal range which was determined in control experiments using differentially labelled normal DNA.

### Comparative expressed sequence hybridisation

CESH analysis was carried out as described previously (Lu *et al*, 2001) using the same capture system and software as for CGH analysis. Five good quality metaphases with 320–450 band resolution were analysed for each case using the Quips CGH analysis software (Vysis) to produce an average profile of the fluorescence intensity ratios. Unlike CGH, the CESH ratios of fluorescence frequently change within a small chromosomal region. Misalignment in averaged profiles can reduce resolution and therefore two or three metaphases with less condensed chromosomes (around 700 band resolution) were also examined individually to precisely define chromosome bands containing differentially expressing genes.

### Data analysis

Gain and loss of chromosomal regions from CGH and gene over- and underexpression from CESH analysis were recorded in Excel spreadsheet format. For each chromosomal band, gain/overexpression was indicated by 1, loss/underexpression by −1 and no change by 0. From this the frequency of aberrations could be calculated. The data, which were subject to unsupervised clustering analysis, were collected using Clustan (http://www.clustan.com). The data were also subject to supervised learning method PAM (prediction analysis for microarray) (Tibshirani *et al*, 2002), which was used to identify expression pattern associated with specific histology. This is carried out by a penalised *t*-statistic, which ranks the elements and a soft-thresholding to identify the set of overexpress regions for classification. To analyse correlations between copy number and expression data the Spearman's rho test was used.

## RESULTS

### Identification of regions of frequent copy number and expression alteration

**Figure 1:**
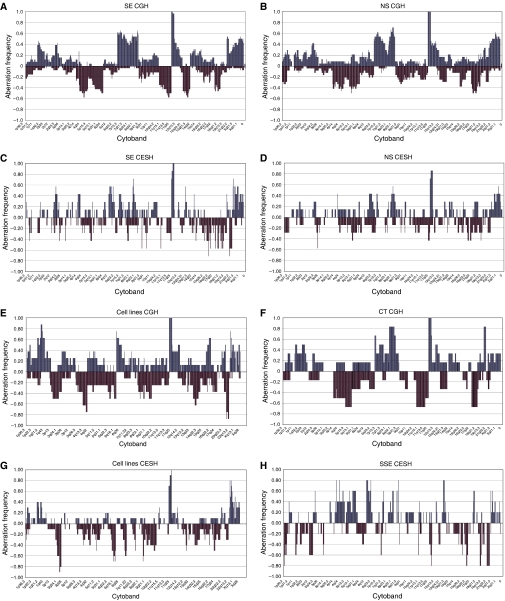
Frequency plots of copy number and expression imbalances in the different subtypes. The frequency of copy number and expression aberrations (*X*-axis) is plotted against the chromosomal location (*Y* axis) for each subtype. Graphs show the proportion of samples, which have copy number or expression aberrations where a positive frequency is equal to proportion of samples with increased copy number or expression and negative frequency equal to the proportion of samples with decreased copy number or expression at each chromosome cytoband. 26 SE, 24 NS, 6 CT and 7 cell lines were analysed by CGH. 7SE, 7NS, 10 cell lines and 5 SSE were analysed by CESH. (**A**), (**B**), (**E**) and (**F**) are the frequency plots for copy number aberrations determined by CGH for SE, NS, TGCT cell lines and combined tumours, respectively. (**C**), (**D**), (**G**) and (**H**) are the frequency plots for expression changes determined by CESH for SE, NS, TGCT cell lines and SSE, respectively.

26 SE-, 6 CT-, 24 NS- and 7 NS-derived cell lines were investigated by metaphase CGH. 7 SE-, 1 CT-, 7 NS- and 9 NS-derived cell lines, 1 SE-derived cell line and 5 SSE were investigated by CESH. Metaphase CGH and CESH data were divided based on subtype and the frequency of aberrations at each chromosomal region was calculated. Frequency plots of imbalances can be seen in Figure 1. Regions of copy number and expression imbalance, which had a frequency of greater than 40% were identified. This identified 42 regions of copy number and 50 regions of expression in addition to the 12p in SE-, NS-, NS-derived cell lines. Analysis of SSE CESH data determined 43 regions of expression imbalance. Comparison between the copy number and expression data highlighted the importance of 20 regions denoted in Table 1. These regions had overlapping copy number and expression imbalances in SE, NS or TGCT cell lines. All identified regions of copy number and expression imbalance can be seen in Supplementary Tables 2 (CGH data) and 3 (CESH data)(http://www.crukdmf.icr.ac.uk/array/array.html).

### Identification of regions that are significantly different between tumour subtypes

**Figure 2:**
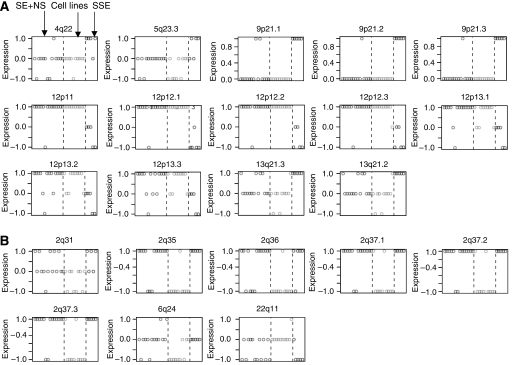
Supervised learning of CESH data. (**A**) Loci that are differentially expressed between TGCT and SSE. (**B**) Loci that are differentially expressed between primary tumours and cell lines. Each box plot represents a chromosome locus identified as discriminatory between the groups, separated by dashed lines. Group 1 (left): primary SE and NS samples; Group 2 (middle): NS-derived cell lines; Group 3 (right): primary SSE samples. Each circle represents a sample within that group. The axis indicates the expression of each sample where 1 denotes increased expression; 0 normal and −1 decreased expression.

Supervised learning of the SE, CT, NS and cell lines CGH data found that loss of two regions at 22q11–q13.3 and 2q14.1–q31 were associated with the cell lines. No other differences were found or could be determined with unsupervised clustering. Prediction analysis of the CESH data could not find significant differences between the SE and NS. However, differences in expression patterns between the primary TGCT (SE and NS), cell lines and SSE were found (Figure 2). These included 12p11–p13.3, which was overexpressed in SE, NS and cell lines but not in SSE. The chromosome bands 2q31, 2q35, 2q36 and 2q37 were associated with overexpression in SE, NS and SSE but underexpression in cell lines. Overexpression from 4q22 and 9p21 was associated with SSE.

### Relationship between copy number and expression in SE and NS

To determine any relationship between the copy number and expression alterations correlations between the parallel CGH and CESH data were tested using Spearman's rho test. All the primary tumours and cell lines except one NS primary sample showed a significant correlation. The Spearman's rho correlation coefficients ranged from 0.089 to 0.719 with an average of 0.267. There was no significant difference in the strength of the correlation between the subtypes or their differentiation status using the student's *t*-test. The data analysis revealed no difference in the association of copy number and expression changes between regions of gain or loss.

## DISCUSSION

Our analysis of copy number and associated differential expression highlights regions likely to be involved in TGCT development. It also illustrates molecular similarities and differences between types and subtypes of these tumours. We identified three novel regions (2q31, 18p11.2 and 21q11) of copy number and expression change in SE and NS. We also defined differential expression to single cytogenetic bands within a number of regions previously characterised with frequent copy number changes.

Three regions showed copy number and expression changes at a high frequency that were consistent with our previous array CGH study of TGCT (McIntyre *et al*, 2004). These regions are 7p22 (0.87–2.02 Mb), 8q21 (86.46–90.69 Mb) and Xq22 (101.54–101.71). Overexpression from 8q21 was found at particularly high frequency involving 71% of SE and 57% of NS. The 8q21 region is frequently gained and amplified in other tumour types, including breast, lung and ovarian cancer (Balleine *et al*, 2000; Byrne *et al*, 2005; Zhu *et al*, 2007). Tumour protein D52 isoform 2 (TPD52) encoded by a gene at 8q21.1 is described as the target for amplification of this region in breast and ovarian cancers (Balleine *et al*, 2000; Byrne *et al*, 2005). Mining of published expression microarray data for TGCTs (Sperger *et al*, 2003) revealed increased expression of *TPD52* in TGCT compared to normal testis with an average fold increase of 3.2 and represents a good candidate for involvement in TGCT. Other differentially expressed genes in regions of imbalance were identified including cell division cycle associated 7 (*CDCA7*) localised to 2q31, which had an average 4.9-fold increase in expression compared with normal testis. CDCA7 is a downstream target of MYC and is frequently overexpressed in human cancers (Osthus *et al*, 2005).

Prediction analysis using the regions of differential expression in TGCT subtypes identified discriminating regions in the SSE, TGCT samples and cell lines. These regions included known differences of 12p overexpression associated with TGCT (Rodriguez *et al*, 2003) and 9p21 associated with SSE (Looijenga *et al*, 2006). Novel differences included overexpression from 4q22 and 5q23.2 associated with SSE. Prediction analysis also highlighted differences between TGCT cell lines and primary tumours at both the copy number and expression levels. These differences may reflect their adaptive response to *in vitro* culturing and limit their usefulness as models of TGCT. However, the majority of regions found in primary tumour material were also found in the cell lines (Table 1; Supplementary Tables 2 and 3.

Our analysis could not find any difference at the genomic or expression level between the SE and NS studied. This provides further evidence that the two subtypes have a similar genetic pathogenesis. Consistent with this is the high incidence of combined tumours (∼10%), which contain elements of both subtypes, and evidence supporting development of NS from SE (Raja *et al*, 1992). Many regions of differential expression were similar in the SSE and the TGCT of adults and adolescents. This may reflect the male germ cell origin of these tumours, which is proposed to differ only in differentiation status between the SSE and TGCT (Oosterhuis and Looijenga, 2005).

Although variable correlation coefficients were found between copy number and expression imbalances, there was a positive correlation in nearly all cases. This indicates that copy number imbalances have a strong effect on the expression patterns and are a major mechanism for aberrant expression in the majority of these tumours. No significant differences were identified in the strength of the correlations between expression and genomic copy number in the different subtypes and in differentiated *vs* non-differentiated tumours. This may suggest that the total effect on expression levels of mechanisms other than chromosomal imbalances is similar in the different subtypes of TGCT. This is despite reported differences in the frequency of CpG island methylation of 0.08% in SE compared to 1.11% in NS (Smiraglia *et al*, 2002; Zhang *et al*, 2005).

A number of regions of copy number change were not associated with an expression change. Similarly, a number of regions were found to have expression changes, but no copy number changes. Similar patterns were found in a study of the metaphase CGH data and expression array data for the preinvasive stage of TGCT of adults and adolescents (Almstrup *et al*, 2005). Many of the genes found overexpressed without associated aberrant copy number are involved in maintenance of pluripotency (e.g. POU5F1 and NANOG) and are also highly expressed in PGCs (Almstrup *et al*, 2005). This is likely to reflect the embryonic stem cell-like nature of these tumours rather than a *de novo* change associated with development of the tumour.

The results presented here highlight correlations between genomic changes and global expression patterns, which suggests that copy number changes play a major role in the expression levels of genes from particular loci in TGCT. Expression patterns showed both similarities and differences at specific regions in TGCT, SSE and derived cell lines. This likely reflects their genetic pathogenesis and common cellular origin. We have defined a number of chromosome regions with a high frequency of both copy number and expression change in TGCT. These require further investigation to implicate oncogenes and tumour suppressor genes in the development of these tumours.

## Figures and Tables

**Table 1:** Regions of overlapping copy number and expression imbalance with a frequency of greater than 40%

**CGH**	**Frequency**	**CESH**	**Frequency**	**CGH**	**Frequency**	**CESH**	**Frequency**
*SE gain*				*SE loss*			
2q31	42	2q31	43	11q12−q25	58	1q24−q25	43
7p22–q36	65	7p15.3–p13	43	18p11.32–p11.2	46	18p11.2	57
8p23.2–q24.3	65	8q21	71	18q12.3−q23	45	18q21.1−q21.3	57
12p13.3–q13.3	100	12p12.3–p11	100				
21q11–q22.3	54	21q11	43				
Xp22.3–p22.1	45	Xp21.3	71				
Xp11.4–p11.3	42	Xp11.4–p11.3	57				
Xp13.3–q28	54	Xp13.2−q21.1	57				
							
*NS gain*				*NS loss*			
7p22–q36	63	7p21.1–p21.2	43	5q14−q21.1	50	5q13.1−q13.3	43
8q12–q24.3	70	8q21	57				
12p13.3–p11	100	12p13.1–p11	86				
Xp11.4–q28	63	Xp11.22–p11.21	43				
Xp11.4–q28	54	Xq22.1	57				
							
*Cell line gain*				*Cell line loss*			
1q21.3–q44	88	1q21.2−q22	44	2q14.1−q36	50	2q36	90
1q21.3–q44	88	1q32.1−q32.2	44	4q25−q35	75	4q28	44
12p13.3–q24.33	100	12p12.3–p11	100	6q25−q27	50	6q26	70
Xp21.3	75	Xp21.3	80	9q31−q33	57	9q31	70
Xp11.4–q27	63	Xp11.4	80	10q11−q22.3	50	10p12.3–p11	44
				15q11−q24	63	15q21−q23	40
				18q21.2−q23	50	18q21.1−q21.3	56

CESH=comparative expressed sequence hybridisation; CGH=comparative genomic hybridisation; SE=seminoma; NS=non-seminoma. 26 SE, 24 NS and 7 cell lines were analysed by CGH. 7SE, 7NS and 10 cell lines were analysed by CESH.
